# Socioeconomic Status Associated With Urinary Sodium and Potassium Excretion in Japan: NIPPON DATA2010

**DOI:** 10.2188/jea.JE20170253

**Published:** 2018-03-05

**Authors:** Naoko Miyagawa, Nagako Okuda, Hideaki Nakagawa, Toshiro Takezaki, Nobuo Nishi, Naoyuki Takashima, Akira Fujiyoshi, Takayoshi Ohkubo, Aya Kadota, Tomonori Okamura, Hirotsugu Ueshima, Akira Okayama, Katsuyuki Miura

**Affiliations:** 1 Department of Public Health, Shiga University of Medical Science, Shiga, Japan; 2 Department of Health and Nutrition, University of Human Arts and Sciences, Saitama, Japan; 3 Medical Research Institute, Kanazawa Medical University, Ishikawa, Japan; 4 Department of International Island and Community Medicine, Kagoshima University Graduate School of Medical and Dental Sciences, Kagoshima, Japan; 5 International Center for Nutrition and Information, National Institute of Health and Nutrition, National Institutes of Biomedical Innovation, Health and Nutrition, Tokyo, Japan; 6 Department of Hygiene and Public Health, Teikyo University School of Medicine, Tokyo, Japan; 7 Center for Epidemiologic Research in Asia, Shiga University of Medical Science, Shiga, Japan; 8 Department of Preventive Medicine and Public Health, Keio University School of Medicine, Tokyo, Japan; 9 Research Institute of Strategy for Prevention, Tokyo, Japan

**Keywords:** urine, sodium, potassium, sodium-to-potassium ratio, socioeconomic status

## Abstract

**Background:**

Although socioeconomic status (SES) may affect food and nutrient intakes, few studies have reported on sodium (Na) and potassium (K) intakes among individuals with various SESs in Japan. We investigated associations of SES with Na and K intake levels using urinary specimens in a representative Japanese population.

**Methods:**

This was a cross-sectional study of 2,560 men and women (the NIPPON DATA2010 cohort) who participated in the National Health and Nutrition Survey Japan in 2010. Casual urine was used to calculate estimated excretion in 24-hour urinary Na (E24hr-Na) and K (E24hr-K). The urinary sodium-to-potassium (Na/K) ratio was calculated from casual urinary electrolyte values. An analysis of covariance was performed to investigate associations of aspects of SES, including equivalent household expenditure (EHE), educational attainment, and job category, with E24hr-Na, E24hr-K, and the Na/K ratio for men and women separately. A stratified analysis was performed on educational attainment and the job category for younger (<65 years) and older (≥65 years) participants.

**Results:**

In men and women, average E24hr-Na was 176.2 mmol/day and 172.3, average E24hr-K was 42.5 and 41.3, and the average Na/K ratio was 3.61 and 3.68, respectively. Lower EHE was associated with a higher Na/K ratio in women and lower E24hr-K in men and women. A shorter education was associated with a higher Na/K ratio in women and younger men, and lower E24hr-K in older men and women.

**Conclusion:**

Lower EHE and a shorter education were associated with a lower K intake and higher Na/K ratio estimated from casual urine specimens in Japanese men and women.

## INTRODUCTION

Cardiovascular diseases (CVD) are one of the most prevalent causes of death worldwide, and hypertension is the main risk factor for CVD.^1^ Excessive salt (sodium chloride) intake and insufficient potassium intake are known to cause hypertension.^2^^–^^4^ Furthermore, the combined effects of sodium and potassium intakes also influence blood pressure and CVD.^5^^,^^6^ The characteristics of individuals who consume large amounts of salt and less potassium need to be clarified in more detail and may lead to the effective prevention of hypertension and CVD in a population. Previous findings, mainly obtained from Western countries, showed that individuals with a lower socioeconomic status (SES)—a lower income or expenditure, lower education attainment, and manual occupations—had greater intakes of meat fat, artificial sugar, and salt, whereas those with a higher SES had greater intakes of vegetables and fruits and a lower intake of sodium.^7^^–^^13^ However, few studies have examined these associations in Japan.^14^^–^^16^

Quantitative assessments of habitual sodium intake using dietary surveys are considered to be challenging for several reasons: the difficulties associated with estimating sodium contents in manufactured foods and dishes served in restaurants, large differences in preferences for a salty taste among individuals, and uncertainty regarding sodium contents in food databases to be used for nutrient calculations.^17^^,^^18^ Although repeated measurements of sodium excretion in urine over 24 hours are regarded as the gold standard, 24-hour urine collection is inconvenient for free-living participants and cannot be easily adapted for large-scale studies. In epidemiological studies, formulae were developed to obtain estimated 24-hour urinary sodium and potassium excretion using casual urinary electrolytes. Previous studies reported relationships of the estimated excretion of 24-hour urinary sodium (E24hr-Na) and potassium (E24hr-K) and the sodium-to-potassium (Na/K) ratio in casual urine samples with blood pressure levels.^19^^–^^21^

To the best of our knowledge, two studies using dietary surveys in Japan found an association between a lower household income or equivalent household expenditure (EHE) and higher salt intake; however, no other aspects of SES were examined. Associations among various socioeconomic factors and sodium and potassium intake levels using urine specimens have not yet been examined in Japan. In the present study, we investigated associations among various socioeconomic factors, including educational attainment, job category, E24hr-Na and E24hr-K levels, and the casual urinary Na/K ratio, among a nationwide Japanese population.

## METHODS

### Study population

In 2010, a prospective cohort study on CVD, the National Integrated Project for Prospective Observation of Non-communicable Disease and its Trends in the Aged 2010 (NIPPON DATA2010), was established.^22^ The study was performed among participants the National Health and Nutrition Survey of Japan in November 2010 (NHNS2010) and the Comprehensive Survey of Living Conditions in June 2010 (CSLC2010), which were conducted by the Ministry of Health, Labour and Welfare of Japan. The details of NHNS2010 and CSLC2010 have been described elsewhere.^23^^–^^26^ In November 2010, 8,815 residents aged 1 year and older from 300 randomly selected districts throughout Japan participated in the dietary survey of NHNS2010. Among 7,229 participants aged 20 years and older, 3,873 (1,598 men and 2,275 women) participated in the full survey of NHNS2010 with a blood test. Among them, 2,898 (1,239 men and 1,659 women) agreed to participate in the baseline survey of NIPPON DATA2010, which included an electrocardiographic analysis, urinalysis, and a questionnaire on CVD. Trained interviewers obtained informed consent before study enrollment. The Institutional Review Board of Shiga University of Medical Science (No. 22–29, 2010) approved this study. Of the 2,898 participants, 91 were excluded because it was not possible to merge data from NHNS2010 or CSLC2010 with NIPPON DATA2010 baseline data, and 247 were excluded because of missing data on urinary data, the employment status, length of education, household expenditure, body mass index (BMI), or marital and living statuses. The remaining 2,560 participants (1,110 men and 1,450 women) were included in the present study.

### Outcomes

Casual urine specimens were obtained when participants visited the designated facilities of NHNS2010. All specimens were kept refrigerated and sent to a central laboratory (SRL, Tokyo, Japan). Urinary creatinine concentrations were measured using an enzymatic method, while sodium and potassium concentrations were measured using selective ion electrode methods. The casual urinary concentration of sodium (mmol/L) was divided by that of potassium (mmol/L) to obtain the Na/K ratio (mol/mol). E24hr-Na and E24hr-K were calculated using Tanaka’s formulae.^27^

### SES

Information on SES was collected from self-administered questionnaires for NHNS2010 (employment status), CSLC2010 (monthly EHE of May 2010, the month before CSLC2010), and NIPPON DATA2010 (length of education). EHE was calculated as household expenditure divided by the square root of the number of family members. Participants were categorized into sex-specific quintiles (Q) of EHE. The employment status was categorized into four groups using the occupational classification of NHNS.^25^ The groups were as follows: group 1, workers for agriculture, forestry, and fishery; group 2, workers for factories and hard labor (ie, construction industries); group 3, clerical, sales, and other service workers, including administrative and professional jobs; and group 4, not working, including homemakers. Educational attainment was categorized into three groups using the length of education (<10, 10–12, and 12 years or over).

### Statistical analysis

The characteristics of study participants are presented as the mean and standard deviation (SD) for continuous variables and as a number and percentage for categorical variables. The association between SES and each outcome was assessed using an analysis of covariance adjusted for age (<30, 30–39, 40–49, 50–59, 60–69, 70–79, or 80 years or older), BMI, residential area (according to the place of residence in NHNS^25^: Hokkaido, Tohoku, Kanto I, Kanto II, Hokuriku, Tokai, Kinki [combined Kinki I and Kinki II], Chugoku and Shikoku, Northern Kyushu, or Southern Kyushu), marital and living statuses (married, unmarried [including never married, divorced, and widowed] and not living alone, or unmarried and living alone), and the type of house (owned or rented: in analyses of EHE only). Since salt consumption has traditionally been higher in northern Japan than in southern or western Japan, the residential area was used as a covariate. Marital and living statuses were obtained using the questionnaire for NIPPON DATA2010. The type of house (owned or rented) was obtained using the questionnaire for CSLC2010 and was used as a covariate as EHE, including house rent, but not a mortgage. The Na/K ratio showed a positively skewed distribution; therefore, we obtained geometric means and geometric 95% confidence intervals (CI) of the Na/K ratio.

Since age has interactions with the relationship between the length of education, occupation groups, and urinary electrolytes, we performed an analysis of covariance in participants aged less than 65 years and 65 years or older separately. The results of the analysis of covariance are reported as the least-squares means with corresponding 95% CIs. Tests for trends were performed with a generalized linear model using variables with ordinal scores assigned to each SES category. *P* < 0.05 was considered to be significant. All statistical analyses were performed using SAS version 9.4 for Windows (SAS Institute Inc., Cary, NC, USA).

## RESULTS

### Participant characteristics

Table 1 shows the characteristics of study participants by sex. The mean age of participants was 60.1 years in men and 58.1 years in women, and 1,450 participants (56.6%) were women. No significant differences were observed in the mean age across Q of EHE in women, whereas men with the highest EHE were older (59.5 years for Q1 vs 61.1 years for Q5) (eTable 1). Participants with a shorter education were older for men (69.7 years) and women (68.5 years). Participants who worked in agriculture, forestry, and fishery (group 1) were also older than those in other job categories (groups 2 and 3) for men and women. The average age of unemployed participants, including homemakers, was younger in women (63.5 years) than in men (70.7 years). Unemployed women were younger (63.5 years) than those working in the group 1 category (66.7 years).

**Table 1.** Characteristics of participants: NIPPON DATA2010 (*n* = 2,560)

	Men (*n* = 1,110)	Women (*n* = 1,450)
Age group, %		
<30	4.1	4.1
30–39	9.0	14.0
40–49	10.3	11.2
50–59	15.8	17.2
60–69	30.7	26.6
70–79	22.7	20.3
80 years or older	7.5	6.5
Residential area, %		
Hokkaido	3.7	4.2
Tohoku	8.6	10.1
Kanto I	15.1	18.3
Kanto II	8.3	7.6
Hokuriku	3.7	5.8
Tokai	7.8	11.6
Kinki	10.9	15.5
Shikoku, Chugoku	7.5	10.9
Northern Kyushu	5.4	8.6
Southern Kyushu	5.6	7.4
Length of education, %		
<10 years	24.5	23.7
10–12 years	42.5	46.6
13 years or over	33.0	29.7
Marital and living statuses, %		
Married	81.7	74.1
Single, not living alone	9.5	14.6
Single, living alone	8.7	11.4
Occupational groups, %		
Group 1	9.0	3.4
Group 2	11.8	3.5
Group 3	42.6	34.6
Group 4	36.6	58.6

Age, years, mean (SD)	60.1 (15.3)	58.1 (15.9)
Body mass index, kg/m^2^, mean (SD)	23.9 (3.2)	22.6 (3.5)
Estimated 24-hour urine sodium excretion, mmol/24h, mean (SD)	176.2 (38.7)	172.3 (38.3)
Estimated 24-hour urine potassium excretion, mmol/24h, mean (SD)	42.5 (7.8)	41.3 (8.1)
Casual urine Na/K ratio, mol/mol, mean (SD)	3.61 (3.49–3.74)	3.68 (3.58–3.79)

Na/K, the sodium-to-potassium ratio.

Geometric means (geometric 95% confidence interval [CI]) are presented for the Na/K ratio.

Occupational group 1: workers for agriculture, forestry, and fishery; group 2: workers for factories and hard labor; group 3: clerical, sales, and other service workers, including administrative and professional jobs; and group 4: not working, including homemakers.

In men and women, the means of E24hr-Na (mmol/24hr) were 176.2 and 172.3, respectively, while corresponding values of E24hr-K were 42.5 and 41.3. The geometric means of the Na/K ratio were 3.61 and 3.68 for men and women, respectively.

### Associations between SESs and urinary sodium and potassium

Table 2 shows the multivariable-adjusted means of urine electrolytes according to the quartile of EHE in men and women. There were no significant associations between EHE and E24hr-Na for men or women. As EHE increased, E24hr-K (mmol/24hr) became higher in men (41.0 for Q1 vs 43.7 for Q5, *P* for trend = 0.007) and women (39.6 vs 41.4, *P* for trend = 0.002). The casual Na/K (mol/mol) ratio was lower for higher quartiles of EHE in women (3.98 for Q1 vs 3.35 for Q5: *P* for trend < 0.001).

**Table 2.** Multivariable-adjusted means of estimated 24-hour urine sodium and potassium and casual urine Na/K according to quintiles of equivalent household expenditure by sex: NIPPON DATA2010

	Estimated 24-hour urine sodium (mmol/24h)			Estimated 24-hour urine potassium (mmol/24h)			Casual urine Na/K (mol/mol)		
	Adjusted mean (95% CI)	*P* value^a^	*P* value^b^	Adjusted mean (95% CI)	*P* value^a^	*P* value^b^	Adjusted mean (95% CI)	*P* value^a^	*P* value^b^
Men									
Q1 (low)	172.3 (166.4–178.2)	0.033	0.887	41.0 (39.8–42.2)	0.003	0.007	3.65 (3.35–3.99)	0.009	0.109
Q2	180.1 (174.0–186.1)			42.1 (40.9–43.3)			3.93 (3.59–4.30)		
Q3	168.5 (162.3–174.8)			41.4 (40.1–42.6)			3.41 (3.11–3.75)		
Q4	172.3 (166.3–178.4)			42.0 (40.8–43.3)			3.48 (3.18–3.81)		
Q5 (high)	173.3 (167.1–179.5)			43.7 (42.5–45.0)			3.26 (2.97–3.57)		
Women									
Q1 (low)	171.6 (166.7–176.6)	0.385	0.357	39.6 (38.6–40.7)	0.008	0.002	3.98 (3.69–4.28)	0.006	<0.001
Q2	169.2 (164.1–174.3)			40.2 (39.1–41.3)			3.73 (3.45–4.02)		
Q3	167.9 (162.9–172.8)			40.6 (39.5–41.6)			3.54 (3.29–3.82)		
Q4	172.5 (167.5–177.5)			41.8 (40.7–42.9)			3.58 (3.32–3.86)		
Q5 (high)	167.5 (162.5–172.6)			41.4 (40.3–42.5)			3.35 (3.10–3.61)		

CI, confidence interval; Na/K, the sodium-to-potassium ratio.

Least-squares means are adjusted for age, residential area, marital and living statuses, body mass index, and the type of house.

Range of quintiles are <8.2, 8.5–11.2, 11.3–14.4, 14.4–18.0, and >18.1 in men, <8.2, 8.5–11.5, 11.5–14.4, 14.5–18.5, and >18.8 in women.

Log-transformed Na/K was used in the analysis of covariance. Reverse transformed values of the results are presented.

^a^Differences were evaluated using an analysis of covariance.

^b^Differences were evaluated using a trend analysis.

The adjusted means of urine electrolytes according to length of education in men and women by age category are shown in Table 3. In younger men, E24hr-Na was lower for those with a longer education (<10 years: 191.0 vs ≥13 years: 175.1, *P* for trend = 0.002). In contrast, E24hr-K increased in those with a longer education in older men and women (<10 years: 41.4 vs ≥13 years: 43.9, *P* for trend = 0.004 in men; <10 years: 39.9 vs ≥13 years: 42.2, *P* for trend = 0.017 in women). Na/K ratios (mol/mol) were also significantly lower in those with a longer education, in younger and older women and in younger men. The Na/K ratios for a shorter education (<10 years) versus longer education (≥13 years) were as follows: 3.75 versus ≥3.23 in younger women; 3.77 versus 3.13 in older women; and 4.56 versus 3.56 in younger men.

**Table 3.** Multivariable-adjusted means of estimated 24-hour urine sodium and potassium and casual urine Na/K according to the length of education by sex and age: NIPPON DATA2010

	Number	Estimated 24-hour urine sodium (mmol/24h)			Estimated 24-hour urine potassium (mmol/24h)			Casual urine Na/K (mol/mol)		
		Adjusted mean (95% CI)	*P* value^a^	*P* value^b^	Adjusted mean (95% CI)	*P* value^a^	*P* value^b^	Adjusted mean (95% CI)	*P* value^a^	*P* value^b^
Men										
<65 years old										
<10 years	73	191.0 (181.5–200.5)	0.007	0.002	42.1 (40.0–44.1)	0.829	0.554	4.56 (3.95–5.27)	0.004	0.001
10–12 years	278	181.0 (175.0–186.9)			42.5 (41.2–43.7)			3.94 (3.61–4.31)		
13 years or over	252	175.1 (169.2–181.0)			42.7 (41.5–44.0)			3.56 (3.25–3.88)		
≥65 years old										
<10 years	199	166.1 (158.3–173.8)	0.676	0.456	41.4 (39.9–43.0)	0.008	0.004	3.24 (2.89–3.63)	0.321	0.151
10–12 years	194	169.4 (161.0–177.8)			43.5 (41.9–45.2)			3.01 (2.66–3.41)		
13 years or over	114	169.2 (159.8–178.7)			43.9 (42.1–45.8)			2.95 (2.57–3.39)		

Women										
<65 years old										
<10 years	93	174.1 (165.6–182.7)	0.252	0.105	41.1 (39.3–43.0)	0.306	0.130	3.75 (3.29–4.28)	0.010	0.004
10–12 years	397	172.2 (166.8–177.6)			41.6 (40.4–42.8)			3.64 (3.35–3.96)		
13 years or over	369	168.2 (162.7–173.7)			42.4 (41.2–43.6)			3.23 (2.97–3.52)		
≥65 years old										
<10 years	251	169.5 (164.1–174.8)	0.791	0.505	39.9 (38.8–41.0)	0.055	0.017	3.77 (3.49–4.07)	0.034	0.009
10–12 years	278	168.2 (162.6–173.8)			41.3 (40.2–42.5)			3.43 (3.16–3.72)		
13 years or over	62	165.8 (155.5–176.0)			42.2 (40.1–44.4)			3.13 (2.70–3.63)		

CI, confidence interval; Na/K, the sodium-to-potassium ratio.

Least-squares means are adjusted for age, residential area, marital and living statuses, and body mass index.

Log-transformed Na/K was used in the analysis of covariance. Reverse transformed values of the results are presented.

^a^Differences were evaluated using an analysis of covariance.

^b^Differences were evaluated using a trend analysis.

Table 4 shows the adjusted means of urine electrolytes according to occupational groups in men and women by age category. In younger men, some differences according to job categories were observed. The adjusted means of E24hr-Na (mmol/24h) were higher in groups 1 and 2 (193.8 for groups 1 and 2) than in other groups, E24hr-K in group 1 (46.9) was the highest, and the Na/K ratio in group 2 was the highest (4.56 mol/mol). No associations were observed for other sex and age categories.

**Table 4.** Multivariable-adjusted means of estimated 24-hour urine sodium and potassium and casual urine Na/K according to occupation groups by sex and age: NIPPON DATA2010

	Number	Estimated 24-hour urine sodium (mmol/24h)		Estimated 24-hour urine potassium (mmol/24h)		Casual urine Na/K (mol/mol)	
		Adjusted mean (95% CI)	*P* value^a^	Adjusted mean (95% CI)	*P* value^a^	Adjusted mean (95% CI)	*P* value^a^
Men							
<65 years old							
Group 1	41	193.8 (181.7–205.8)	<0.001	46.9 (44.4–49.5)	0.001	3.76 (3.12–4.52)	0.003
Group 2	102	193.8 (186.2–201.5)		43.2 (41.6–44.9)		4.56 (4.05–5.13)	
Group 3	381	175.1 (169.6–180.5)		42.1 (41.0–43.3)		3.68 (3.38–4.00)	
Group 4	79	168.0 (158.7–177.3)		41.1 (39.1–43.0)		3.45 (2.99–3.98)	
≥65 years old							
Group 1	59	168.6 (156.3–180.8)	0.408	42.9 (40.5–45.3)	0.460	3.12 (2.60–3.74)	0.909
Group 2	29	160.6 (145.0–176.2)		41.7 (38.6–44.8)		2.95 (2.34–3.72)	
Group 3	92	162.6 (152.2–172.9)		41.5 (39.4–43.5)		3.01 (2.58–3.51)	
Group 4	327	169.3 (162.2–176.4)		42.9 (41.5–44.3)		3.14 (2.82–3.49)	

Women							
<65 years old							
Group 1	20	185.8 (169.2–202.5)	0.202	44.1 (40.5–47.7)	0.129	3.77 (2.91–4.89)	0.825
Group 2	42	165.7 (154.1–177.3)		40.3 (37.8–42.8)		3.53 (2.95–4.23)	
Group 3	435	170.0 (165.0–175.0)		41.6 (40.6–42.7)		3.48 (3.22–3.77)	
Group 4	362	172.0 (166.1–177.8)		42.5 (41.3–43.8)		3.40 (3.10–3.72)	
≥65 years old							
Group 1	29	161.9 (147.2–176.5)	0.566	39.2 (36.2–42.3)	0.455	3.62 (2.93–4.48)	0.761
Group 2	9	163.0 (137.4–188.6)		37.5 (32.2–42.9)		4.00 (2.76–5.82)	
Group 3	66	173.3 (163.3–183.3)		40.9 (38.8–43.0)		3.77 (3.26–4.36)	
Group 4	487	168.5 (164.1–172.9)		40.8 (39.9–41.8)		3.53 (3.31–3.77)	

CI, confidence interval; Na/K, the sodium-to-potassium ratio.

Least-squares means are adjusted for age, residential area, marital and living statuses, and body mass index.

Occupational group 1: workers for agriculture, forestry, and fishery; group 2: workers for factories and hard labor; group 3: clerical, sales, and other service workers, including administrative and professional jobs; and group 4: not working, including homemakers.

Log-transformed Na/K was used in the analysis of covariance. Reverse transformed values of the results are presented.

^a^Differences were evaluated using an analysis of covariance.

In the sensitivity analysis, the associations between socioeconomic factors and urine electrolytes were similar when total energy intake per day was used instead of BMI for adjustments.

## DISCUSSION

In the present study on a nationwide survey of the general Japanese population, some aspects of SES were associated with E24hr-Na and E24hr-K, as well as with the casual urine Na/K ratio. Higher EHE was associated with higher E24hr-K in men and women and with a lower urinary Na/K ratio in women. A longer education was associated with higher E24hr-K in older men and women and with a lower urinary Na/K ratio in women and younger men. In younger men, those working in the group 2 category (such as factory workers) had a higher urinary Na/K ratio and E24hr-Na.

In previous studies on Western^7^^–^^10^ and Asian populations,^13^^,^^15^^,^^28^ a shorter education, lower economic status, and manual labor were associated with a higher sodium intake and lower potassium, fruit, and vegetable intakes. In contrast, a longer education and higher economic status were associated with lower sodium and higher potassium intakes. The results of the present study were consistent with these findings. The World Health Organization examined the factors influencing health in connection with social and economic environments and reported that “a higher income and social status are linked to better health”, while “low education levels are linked to poor health”.^29^ The results of the present study may partly explain the associations between low socioeconomic factors and poor health.

In the present study, E24hr-K was lower for participants with lower EHE than those with higher EHE (Table 2). Fruit and vegetables are major sources of dietary potassium in Japan.^25^^,^^30^ These foods are low in energy density and are expensive for households with a limited budget. NHNS2010 showed that vegetable intake was significantly greater with an annual household income of more than 6,000,000 Japanese yen (JPY) than with a lower income.^25^ Our results suggested that participants with higher EHE consumed more vegetables and fruits.

The present results showed that a longer education was associated with a lower Na/K ratio (Table 3). Lower E24hr-Na was observed in younger men with a longer education, and higher E24hr-K for older men and women with a longer education. The permeation of salt reductions and purchasing power of various foods, including vegetables and fruits, among those with a longer education may have caused differences in the urinary Na/K ratio.

Regarding participants in the group 1 job category (such as agriculture and fishery), E24hr-Na and E24hr-K were both higher in younger men than those in group 3 (clerical and service workers) and unemployed (group 4) participants. These groups may have consumed more food because of their higher physical activity at work and easy access to foods, including the vegetables they grow.

We examined the influence of EHE, educational attainment, and job category on the urinary excretion of sodium and potassium separately, and the results obtained were consistent with previous findings. These socioeconomic factors interact with one another. The distributions of two other socioeconomic factors from the viewpoint of one factor are shown in eTable 1, eTable 2, and eTable 3. Many associations between socioeconomic factors could be observed, such as more participants with longer education years engaged in clerical and service work.

In Japan, prominent gender differences have also been observed in socioeconomic factors.^31^ For example, the percentage of part-time employment is higher among women than men. Furthermore, the purchasing power of a household may be affected more by the husband’s than by the wife’s income because of the lower average wage for women than men. The employment rate of women is lower, even for those with a high education background. These factors may have weakened the associations between the job category and urinary sodium and potassium excretion in women.

This is the first study to demonstrate the associations between socioeconomic factors and sodium and potassium intake levels estimated using casual urine obtained in a nationwide survey from a Japanese general population. The quantitative evaluation of sodium intake using dietary surveys involves many challenges, including the possibilities of incorrect dietary records, recall bias, and insufficient food composition databases. The intakes of manufactured food and restaurant food are increasing in Japan. Since sodium contents in restaurant or prepared food vary markedly according to restaurants and food companies, the difficulties associated with estimating sodium intake using dietary surveys are increasing. Estimations using urine specimens are expected to increase in importance. Although estimated sodium and potassium excretion values from a casual urine sample are not sufficient to estimate individual 24-hr urinary excretion, it is appropriate for obtaining representative values for a population. The associations among groups categorized by socioeconomic factors and urinary sodium and potassium excretion in the present study are considered to be reliable.

There are several limitations in the present study. Due to the cross-sectional nature of this study, we need to be conservative when assessing a causal relationship between socioeconomic factors and urinary sodium and potassium excretion. Furthermore, we estimated 24-hour urinary sodium and potassium using casual urine. A discrepancy may exist between estimated sodium excretion and actual 24-hour urine sodium excretion.^27^ In addition, since the occupation status was classified into four categories using the occupational classification in NHNS, its misclassification may have weakened the relationship. Lastly, we cannot exclude residual confounding from unmeasured or unknown factors.

In conclusion, estimated 24-hour urinary potassium excretion was significantly lower and the casual urine Na/K ratio was significantly higher among participants with a lower EHE and shorter education. This association was more evident in men than in women. In younger men, working in factories and manual jobs was also associated with an unfavorable sodium-potassium intake profile. Lower aspects of SES may be important factors associated with the unfavorable sodium-potassium intake profile in a general Japanese population.

## APPENDIX A. SUPPLEMENTARY DATA

eTable 1. Characteristics of study participants according to sex-specific quintiles of equivalent household expenditure: NIPPON DATA2010

eTable 2. Characteristics of study participants according to the length of education by sex and age: NIPPON DATA2010

eTable 3. Characteristics of study participants according to occupational groups by sex and age: NIPPON DATA2010
