# Supplementary material for: Socioeconomic Status Associated With Urinary Sodium and Potassium Excretion in Japan: NIPPON DATA2010
Source: J Epidemiol. 2018 Mar 5;28(Suppl 3):S29–34. doi: 10.2188/jea.JE20170253 (PMC5825693; doi:10.2188/jea.JE20170253)
Supplement: Supplementary file 1 [file je-28-S029-s001.pdf]

eTable 1. Characteristics of study participants according to sex-specific quintiles of equivalent household expenditure: NIPPON DATA2010

	Equivalent household expenditure					*P* value[a]
	Q1 (low)	Q2	Q3	Q4	Q5 (high)	
Men						
Age, years	59.5 (15.6)	58.2 (16.6)	60.2 (15.3)	61.6 (14.0)	61.1 (15.1)	0.045
Body mass index, kg/m^2	23.9 (3.1)	23.7 (3.2)	24.0 (3.3)	24.0 (3.1)	23.9 (3.1)	0.542
Length of education, years	11.5 (2.6)	12.1 (2.7)	12.3 (2.5)	12.5 (2.7)	13.2 (2.6)	<0.001
Occupational group, %						
Group 1	13.0	13.9	7.9	4.7	5.9	0.001
Group 2	14.3	13.9	14.9	8.1	8.1	
Group 3	37.7	40.3	43.3	43.6	48.2	
Group 4	35.0	31.9	34.0	43.6	37.8	
Women						
Age, years	60.8 (16.4)	57.1 (16.0)	56.9 (15.6)	57.1 (15.3)	58.4 (15.8)	0.106
Body mass index, kg/m^2	22.8 (3.8)	22.8 (3.5)	22.9 (3.5)	22.3 (3.2)	22.3 (3.5)	0.028
Length of education, years	11.1 (2.4)	11.8 (2.3)	12.1 (2.0)	12.4 (2.1)	12.5 (2.1)	<0.001
Occupational group, %						
Group 1	6.5	4.4	2.7	2.1	1.4	0.048
Group 2	4.8	4.4	3.7	2.8	2.0	
Group 3	30.6	33.8	36.9	35.8	35.6	
Group 4	58.2	57.5	56.8	59.3	61.0	

Data are expressed as mean (standard deviation [SD]) or percentage of participants.

a Differences were evaluated using a trend analysis (continuous variables) and the chi-squared test (categorical variables).

Occupational group 1: workers for agriculture, forestry, and fishery; group 2: workers for factories and hard labor; group 3: clerical, sales, and other service workers, including administrative and professional jobs; and group 4: not working, including homemakers.

eTable 2. Characteristics of study participants according to the length of education by sex and age: NIPPON DATA2010

	Length of education			P value[a]
	<10 years	10–12 years	13 years or over	
Men, <65 years old				
Age, years	57.3 (9.4)	49.8 (11.7)	46.3 (11.7)	<0.001
Body mass index, kg/m^2	24.3 (3.1)	23.9 (3.2)	24.3 (3.7)	0.523
Equivalent household expenditure	10.4 (5.3)	15.5 (26.5)	15.3 (11.1)	0.167
Occupational group, %				
Group 1	12.3	9.0	2.8	<0.001
Group 2	28.8	20.1	9.9	
Group 3	37.0	57.2	77.4	
Group 4	21.9	13.7	9.9	
Men, ≥65 years old				
Age, years	74.3 (6.4)	71.9 (5.1)	73.1 (6.0)	0.029
Body mass index, kg/m^2	23.7 (2.9)	23.5 (2.8)	23.7 (2.7)	0.862
Equivalent household expenditure	13.4 (8.8)	14.7 (8.0)	17.9 (9.7)	<0.001
Occupational group, %				
Group 1	17.6	9.8	4.4	<0.001
Group 2	9.5	5.2	0.0	
Group 3	10.6	21.1	26.3	
Group 4	62.3	63.9	69.3	
Women, <65 years old				
Age, years	54.2 (11.7)	49.8 (11.7)	44.0 (10.8)	<0.001
Body mass index, kg/m^2	23.9 (4.5)	22.5 (3.6)	21.5 (3.1)	<0.001
Equivalent household expenditure	10.8 (5.7)	15.0 (11.1)	16.2 (14.1)	0.001
Occupational group, %				
Group 1	4.3	3.0	1.1	<0.001
Group 2	7.5	7.1	1.9	
Group 3	28.0	46.9	60.4	
Group 4	60.2	43.1	36.6	
Women, ≥65 years old				
Age, years	73.9 (5.9)	72.6 (5.8)	71.6 (5.4)	0.001
Body mass index, kg/m^2	23.6 (3.6)	22.9 (3.2)	22.9 (2.8)	0.033
Equivalent household expenditure	12.9 (11.8)	14.9 (10.9)	17.9 (10.2)	0.001
Occupational group, %				
Group 1	7.5	3.6	0.0	-
Group 2	1.6	1.8	0.0	
Group 3	9.1	10.7	20.6	
Group 4	80.7	83.2	77.8	

Data are expressed as mean (standard deviation [SD]) or percentage of participants.

a Differences were evaluated using a trend analysis (continuous variables) and the chi-squared test (categorical variables).

Occupational group 1: workers for agriculture, forestry, and fishery; group 2: workers for factories and hard labor; group 3: clerical, sales, and other service workers, including administrative and professional jobs; and group 4: not working, including homemakers.

eTable 3. Characteristics of study participants according to occupational groups by sex and age: NIPPON DATA2010

	Occupational group				*P* value[a]
	Group 1	Group 2	Group 3	Group 4	
Men, <65 years old					
Age, years	55.2 (11.4)	47.6 (11.8)	47.6 (11.6)	56.2 (10.7)	<0.001
Body mass index, kg/m^2	24.5 (3.1)	23.8 (3.2)	24.2 (3.5)	23.9 (3.3)	0.511
Equivalent household expenditure	14.8 (23.1)	11.5 (5.1)	15.8 (22.9)	13.8 (7.2)	0.252
Length of education, years	11.8 (1.8)	12.1 (2.5)	13.6 (2.2)	12.6 (2.5)	<0.001
Men, ≥65 years old					
Age, years	73.5 (6.3)	69.0 (3.4)	70.4 (4.5)	74.2 (6.0)	<0.001
Body mass index, kg/m^2	22.6 (2.8)	24.0 (2.4)	23.7 (2.4)	23.7 (2.9)	0.033
Equivalent household expenditure	12.5 (8.3)	14.9 (6.6)	16.8 (9.9)	14.8 (8.7)	0.036
Length of education, years	10.1 (2.7)	10.0 (1.5)	12.4 (2.5)	11.5 (2.8)	<0.001
Women, <65 years old					
Age, years	56.5 (6.1)	48.1 (9.7)	45.7 (11.5)	49.8 (12.2)	<0.001
Body mass index, kg/m^2	22.9 (3.1)	22.8 (4.1)	21.8 (3.3)	22.6 (3.8)	0.007
Equivalent household expenditure	18.6 (31.5)	12.2 (6.5)	15.4 (13.8)	14.8 (8.1)	0.213
Length of education, years	11.8 (1.6)	11.8 (1.5)	13.2 (1.8)	12.5 (2.0)	<0.001
Women, ≥65 years old					
Age, years	73.8 (5.0)	66.3 (1.4)	69.6 (3.4)	73.6 (6.0)	<0.001
Body mass index, kg/m^2	24.1 (3.6)	22.0 (3.3)	23.8 (3.5)	23.1 (3.3)	0.140
Equivalent household expenditure	9.3 (5.3)	11.5 (4.0)	12.9 (7.3)	14.9 (12.0)	0.035
Length of education, years	9.8 (1.8)	10.7 (1.6)	11.4 (2.1)	10.8 (2.2)	0.009

Data are expressed as mean (standard deviation [SD]) or percentage of participants.

[a] Differences were evaluated using an analysis of variance.

Occupational group 1: workers for agriculture, forestry, and fishery; group 2: workers for factories and hard labor; group 3: clerical, sales, and other service workers, including administrative and professional jobs; and group 4: not working, including homemakers.
